# Novel heterozygous mutation in *COL4A4* responsible for Alport syndrome in a Chinese family

**DOI:** 10.3389/fgene.2022.899006

**Published:** 2022-09-09

**Authors:** Ran Du, Jishi Liu, Yiqiao Hu, Song Peng, Liangliang Fan, Rong Xiang, Hao Huang

**Affiliations:** ^1^ Department of Nephrology, The Third Xiangya Hospital Central South University, Changsha, China; ^2^ Department of Cell Biology, Hunan Key Laboratory of Animal Models for Human Diseases, School of Life Sciences, Central South University, Changsha, China; ^3^ Hunan Key Laboratory of Organ Fibrosis, Central South University, Changsha, China; ^4^ Department of Radiology, The Third Xiangya Hospital Central South University, Changsha, China

**Keywords:** chronic kidney disease, Alport syndrome, COL4A4, whole-exome sequencing, mutation

## Abstract

**Background:** Chronic kidney disease, a global public health problem, results in kidney damage or a gradual decline in the glomerular filtration rate. Alport syndrome is commonly characterized by chronic glomerulonephritis caused by a structural disorder in the glomerular basement membrane. Currently, three disease-causing genes, namely collagen type IV alpha 3–5 (*COL4A3*, *COL4A4*, and *COL4A5*), have been associated with the occurrence of Alport syndrome.

**Methods:** We enrolled a Chinese family where the affected individuals suffered from recurrent hematuria and proteinuria. The proband was selected for whole-exome sequencing to identify the pathogenic mutations in this family.

**Results:** After data filtering, a novel heterozygous *COL4A4* variant (NM_000092: c.853G>A/p. G285A) was identified as the putative genetic lesion in the affected individuals. Further co-segregation analysis using Sanger sequencing confirmed that this novel *COL4A4* mutation (c.853G>A/p. G285A) exists only in the affected individuals and is absent in other healthy family members as well as in the control cohort of 200 individuals from the same locality. According to American College of Medical Genetics and Genomics guidelines, the mutation was classified as ‘potentially pathogenic’. A bioinformatics-based prediction analysis revealed that this mutation is pathogenic and may disrupt the structure and function of type IV collagen. This variant is located at an evolutionarily conserved site of COL4A4.

**Conclusion:** In this study, we identified a novel heterozygous *COL4A4* variant (c.853G>A) in a Chinese AS family and assisted to diagnose this AS proband as autosomal-dominant Alport syndrome (ADAS). Our study expands the spectrum of Alport syndrome mutations and contributes to the genetic counseling and diagnosis of patients with Alport syndrome.

## Introduction

Chronic kidney disease (CKD) is one of the most common renal diseases that present with kidney damage (proteinuria, hematuria, or anatomical abnormality) or a decline in glomerular filtration rate (<60 ml/min/1.73 m^2^ for at least 3 months) (Inker et al., 2014). The CKD incidence rate has recently increased up to 10.8% in China and 14.8% in the United States (Wang et al., 2019; Zhang et al., 2012). As the initial kidney damage progresses to kidney failure, the affected individuals are at an elevated risk of cardiovascular disease and sudden death (Charytan et al., 2016; Wang et al., 2019). Therefore, CKD has become a global public health concern (Wang et al., 2018). CKD can be caused by congenital anomalies in the kidneys and urinary tract, steroid-resistant nephrotic syndrome, chronic glomerulonephritis, renal cystic ciliopathies, and urinary stone disease (Vivante and Hildebrandt,.,2016).

Alport syndrome (AS) is a form of chronic glomerulonephritis characterized by structural disorder of the glomerular basement membrane (GBM). AS is primarily characterized by hematuria and progressive kidney failure, while some affected individuals also exhibit hearing loss and ocular abnormalities (Wu et al., 2021). According to the report of the Japanese Society of Pediatric Nephrology (JSPN) in 2015, the clinical characteristics of AS could divide into three types. Persistent hematuria is the primary feature which is the main criterion of AS. Secondary features always display type IV collagen or GBM abnormal. Accessory features show family history, hearing loss or ocular abnormalities (Nozu et al., 2019). AS has a high prevalence, affecting about from one in 5,000 to one in 53,000 individuals; however, it has a low awareness rate because of its imperceptible clinical phenotype (Gibson et al., 2021). If left untreated, these patients could progress to kidney failure (Kumela et al., 2019). Patients with kidney failure require dialysis for survival while it seldom aids in the recovery; those receiving a kidney transplant may be an exception. However, this procedure becomes a heavy burden for both the hospitals and families (Sharif and Baura., 2018; Connaughton and Hildebrandt., 2020). Therefore, early diagnosis and therapy are crucial for patients with AS.

With the continuous use of genetics in clinics, genetic technology has become a powerful and cost-effective tool for clinical diagnosis and therapy. Mutations in *COL4A3*, *COL4A4*, and *COL4A5* have been detected in patients with AS. *COL4A5* variants account as the causative reasons for more than 85% of AS patients (Namba et al., 2021; Pirson, 1999). In addition, studies have revealed that patients with heterozygous mutations in either *COL4A3* or *COL4A4* have a mild phenotype that causes hematuria and proteinuria and do not suffer from hearing loss or ocular defects (Savige et al., 2003; Fan et al., 2020).

In this study, we investigated a Chinese family suffering from long-term hematuria and proteinuria. Whole-exome sequencing (WES) and Sanger sequencing were performed to explore the genetic lesions in the family.

## Materials and methods

### Ethical compliance

This study was approved by the Ethics Committee of the Third Xiangya Hospital Central South University, Changsha, China and was performed in accordance with the principles outlined enshrined in the Declaration of Helsinki. The patients/participants provided written informed consent to participate in the study.

### Participants/patients

**FIGURE 1:**
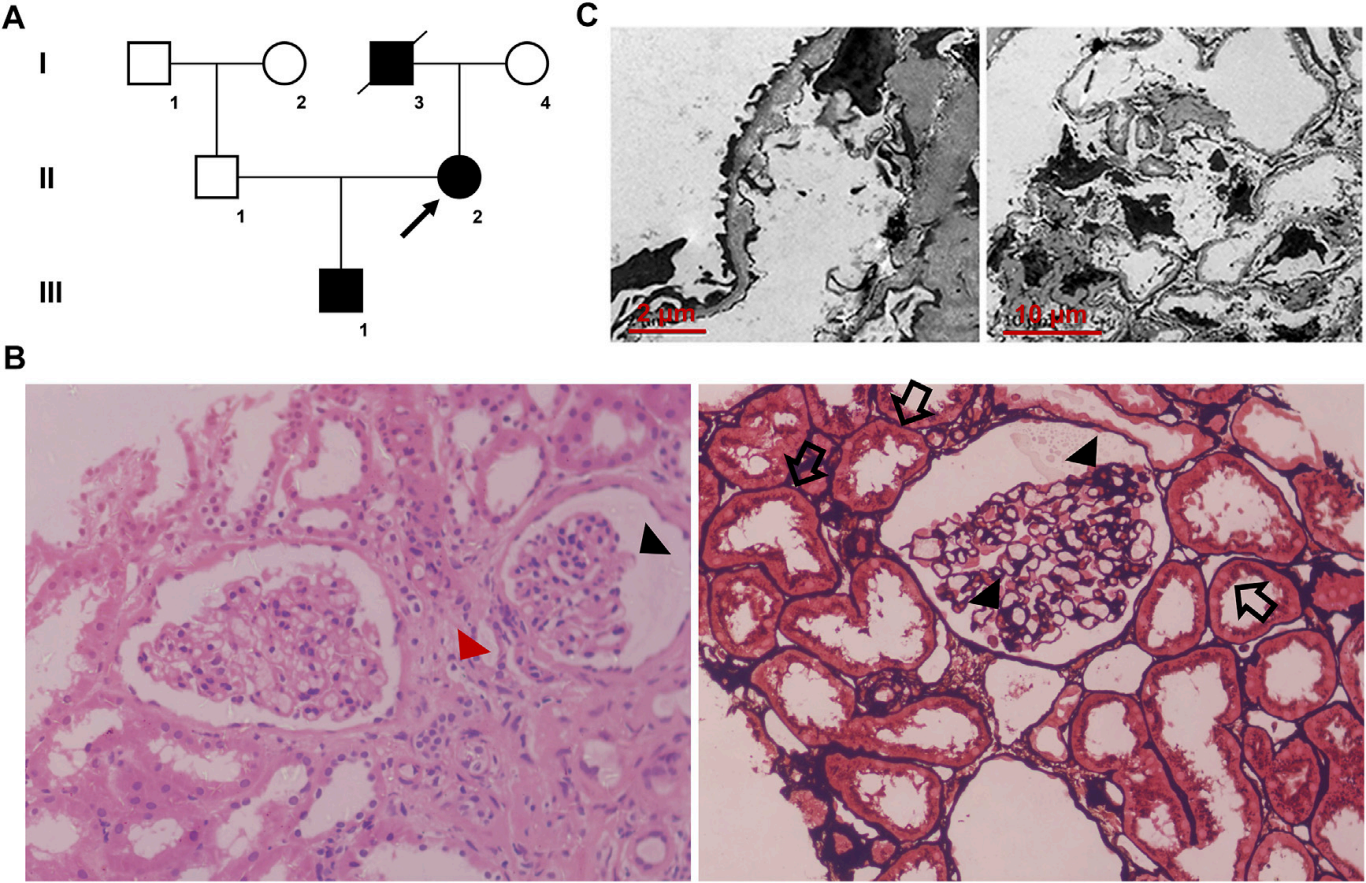
**(A)** Pedigree of the family. Family members are identified by generations and numbers. Squares = men; circles = women; black symbols = individuals with **(C)**853G>A mutation; and arrow = the proband **(B)** Histopathological findings of the proband. Light microscopy images (40×) display focal segmental glomerulosclerosis (black triangles) and swollen or granulated tubular epithelial cells (black arrows), as well as scattered foam cells in the interstitium (red triangle) **(C)** Transmission electron microscopic images display a few round and electron-dense bodies in the mesangial insertion. The glomerular capillary wall is diffusely thickened, and the GBM thickness is irregular with an uneven density. There is characteristic combined diffuse effacement of foot processes. GBM, glomerular basement membrane.

A family that included seven individuals was investigated in this study (Figure 1A). Peripheral blood samples were collected from two affected (III-1 and II-2) and two healthy family members (I-4 and II-1). Clinical data, including renal function and urine testing, were recorded carefully. Renal biopsy and renal pathology of the proband (II-2) were performed using hematoxylin-eosin (HE) and periodic acid-silver metheramine (PASM) staining and transmission electron microscopy (TEM) analysis. In addition, 200 unrelated healthy individuals from the same locality were enrolled as normal controls.

### Whole-exome sequencing

Genomic DNA was extracted using the DNeasy blood and tissue kit (Qiagen, Valencia, CA, United States). Exome capture and next-generation sequencing were conducted at Berry Genomics (Beijing, China). One microgram of quantified genomic DNA from each individual was captured using the SureSelect Human All Exon Kit V6 (Agilent Technologies, Inc., CA, United States) and sequenced using the Illumina HiSeq4000 platform (Illumina Inc., CA, United States). Briefly, the genomic DNA was randomly carved by a Covaris S220 sonicator (Covaris, Inc., MA, United States). The fragmented DNA underwent three enzymatic steps: end repair, A-tailing, and adapter ligation. The adapter-ligated DNA fragments were amplified using Herculase II Fusion DNA Polymerase (Agilent). Finally, the exosomes in the pre-capture libraries were captured using the SureSelect capture library kit (Agilent). After DNA quality assessment, the captured DNA library was used for next-generation sequencing on the Illumina HiSeq4000 platform. Downstream processing was carried out using the Genome Analysis Toolkit (GATK), Varscan2, and Picard, and variant calls were made with the GATK Haplotype Caller 12. Variant annotation referred to Ensemble release 82, and filtering was conducted using ANNOVAR Documentation.

The filtering strategies conformed to those from our previous study (Dong et al., 2021; Wang C et al., 2020;; Huang et al., 2022). Non-synonymous single-nucleotide polymorphisms (SNPs) or frameshift-causing insertion–deletion mutations (INDELs) with an alternative allele frequency >0.005 in the NHLBI Exome Sequencing Project Exome Variant Server (ESP6500), dbSNP1144 (http://www.ncbi.nlm.nih.gov/projects/SNP/index.html), the 1000 Genomes Project (http://www.1000genomes.org/), the ExAC database (http://exac.broadinstitute.org), and in-house exome databases of Berry (2000 exomes) were used for further analysis. The filtered single-nucleotide variant (SNVs) and INDELs were predicted using SIFT (http://sift.jcvi.org/), Polyphen2 (http://genetics.bwh.harvard.edu/pph2/) and MutationTaster (http://www.mutationtaster.org/) to be pathogenic (Wang C. Y et al., 2020). CKD-related genes were used to filter candidate mutations (Vivante and Hildebrandt,.,2016).

### Co-segregation analysis

Co-segregation analysis was performed on each family member using Sanger sequencing. The primer pairs used for PCR amplification were designed using Primer 3 (primer sequences will be provided upon request). The sequences of the PCR products were determined using an ABI 3100 Genetic Analyzer (ABI, Foster City, CA, United States).

## Results

### Clinical description

The proband (II-2), a 32-year-old woman, was admitted to our hospital due to hematuria diagnosed during her health checkup. Laboratory analysis showed the following: 1,355.8/μl (<22.7/μl for normal) erythrocytes and 1,865.9/μl (<130.7/μl for normal) bacteria in urine sediment; microalbuminuria 585.8 mg/L (<20 mg/L for normal); blood urea nitrogen 2.93 mmol/L; blood creatinine 35 μmol/L; uric acid 221 μmol/L; the estimated glomerular filtration rate (eGFR) was 138.7 ml/min/1.73 m^2^. As she description, she got hematuria at the age of 25. A family history survey found that the proband’s father (I-3) died of kidney failure in 65 years old. The proband’s son (III-1) also presented hematuria at the age of 10. The primary diagnosis was chronic nephritis. The latest urine testing result of III-1 showed proteinuria + and hematuria 1+. No hearing or ocular malformations were observed in the proband and other family members. Renal biopsy and pathology testing of the proband (II-2) showed focal segmental glomerulosclerosis, swollen or granulated tubular epithelial cells, and scattered foam cells in the interstitium (Figure 1B). TEM examination revealed that the glomerular capillary wall was diffusely thickened, and the GBM thickness was irregular, with an uneven density (Figure 1C).

The proband (II-2) accepted renal function protecting treatment like taking perindopril tablets regularly and discharged without any complications. We also suggested the proband (II-2) and her affected family members should seek medical treatment when feel a discomfort.

### Genetic analysis

**FIGURE 2:**
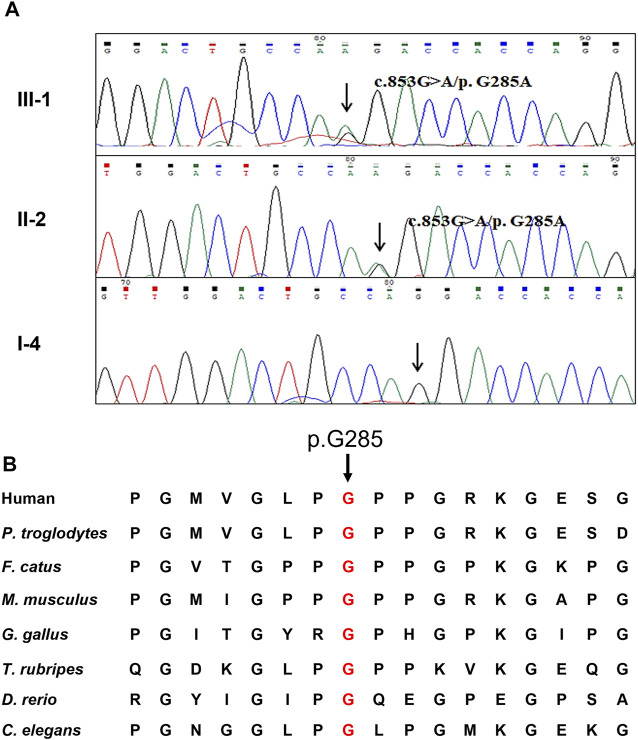
Genetic description of the Chinese family **(A)** Sanger sequencing results of the missense *COL4A4* mutation among two affected family members and one healthy member **(B)** Alignment analysis of this site (p.G285) in COL4A4 amino acid sequences shows that the site (p.285) is highly conserved.

WES yielded 9.61 Gb data with 99.6% coverage of the target region and 99.0% of the target covered over 10×. After data filtering, a *COL4A4* mutation (NM_000092: c.853G>A/p.G285A) was highly suspected to be the genetic lesion in the patient (Figure 2A). No other potential pathogenic mutations known to cause kidney disease were found in the analysis. Further co-segregation analysis revealed that the novel *COL4A4* mutation existed in the affected individual (III-1) and was absent in the two other healthy family members (II-1 and I-4) and the control cohort. Bioinformatics-based prediction revealed that this mutation is pathogenic and may disrupt the structure and function of type IV collagen. The novel mutation (c.853G>A/p.G285A), resulting in the substitution of glycine by alanine, was located at a highly evolutionarily conserved site of the COL4A4 protein (Figure 2B). According to American College of Medical Genetics and Genomics (ACMG) guidelines (Richards et al., 2015; Savige and Harraka et al., 2021), this mutation is likely pathogenic (PM1+PM2+PP1+PP3+PP4).

## Discussion

In this study, a *COL4A4* novel mutation (p.G285A) was identified in the affected individuals of a Chinese family with a history of AS by WES and Sanger sequencing. Our study data was consistent with previous studies in humans and animals, revealing that mutations in *COL4A4* may lead to AS (Fan et al., 2020; Korstanje et al., 2014). Our study further confirmed the clinical diagnosis and proved that genetic analysis could play a pivotal role in the personalized diagnosis of AS (Connaughton and Hildebrandt., 2020).

Depending on the disease-causing gene, AS can be divided into three models: semidominant X-linked due to mutations in *COL4A5* and autosomal-dominant and autosomal-recessive inheritance patterns associated with *COL4A3* and/or *COL4A4* (Shang et al., 2019). Only 172 mutations in *COL4A4* have been reported in the Human Gene Mutation Database, compared to over 1000 *COL4A5* mutations and twice as few as *COL4A3* mutations. In addition, the new AS data revealed an autosomal-dominant pattern in up to 31% of the families (Fallerini et al., 2014). Heterozygous *COL4A3* and *COL4A4* variants are at least 20 times more common than the *COL4A5* variants in the population (Savige et al., 2021). However, only 8% of autosomal-dominant AS cases have been identified (Nozu et al., 2019). These data underscore the importance of further exploring the putative spectrum of AS-causing mutations.

COL4A4 comprises three distinct domains: a short N-terminus, a long central triple-helix with G-X-Y repeats, and a non-collagenous C-terminus (Fan et al., 2020). In this study, the substitution of a hydrophilic amino acid (Gly) with a hydrophobic amino acid (Ala) at position 285 in COL4A4 was identified in the triple-helical domain. A conservative analysis showed that this residue at position 285 is conserved in proteins and is crucial for normal protein function. Substitution of this residue may result in its misincorporation into the triple helix of type IV collagen, and leading to destabilization of the molecular superstructure (Yang et al., 2019; Demir and Caliskan, 2020), which may disrupt the structure and function of collagen in GBM and result in AS.

Chronic renal diseases usually present a low awareness rate as they are rare owing to hidden or a lack of unique symptoms. Therefore, individuals do not seek medical attention until they develop kidney failure (Kumela et al., 2019). AS is a chronic renal disease characterized by hematuria, proteinuria, progressive CKD, and kidney failure (Savige et al., 2016). Unlike X-linked AS that presents with a severe phenotype, AS-causing heterozygous mutations show a mild decline in kidney function. However, up to 10% of the affected individuals gradually develop kidney failure by the age of 60 (Pierides et al., 2009), wherein it is difficult to provide effective treatment. Genetic screening strategies, such as premarital checks or antenatal visits, may be effective in increasing awareness on AS and thereby preventing the likelihood and progression of AS in future generations.

In conclusion, we identified a novel heterozygous *COL4A4* mutation (c.853G>A/p.G285A) in an autosomal-dominant AS family, using WES and Sanger sequencing. The present study on the novel mutation further explains the possible cause of AS and expands the spectrum of AS mutations, thus contributing to the genetic diagnosis and counseling for patients with kidney diseases.

## Data Availability

The data presented in the study are deposited in the GSE-Human repository, accession number HRA002787 (https://ngdc.cncb.ac.cn/gsa-human/browse/HRA002787).
